# Estradiol Replacement at the Critical Period Protects Hippocampal Neural Stem Cells to Improve Cognition in APP/PS1 Mice

**DOI:** 10.3389/fnagi.2020.00240

**Published:** 2020-08-04

**Authors:** Yaoyao Qin, Dong An, Weixing Xu, Xiuting Qi, Xiaoli Wang, Ling Chen, Lei Chen, Sha Sha

**Affiliations:** ^1^Department of Physiology, Nanjing Medical University, Nanjing, China; ^2^State Key Lab of Reproductive Medicine, Nanjing Medical University, Nanjing, China

**Keywords:** Alzheimer’s disease (AD), β-amyloid (Aβ), estradiol (E2), hippocampus, neurogenesis

## Abstract

It has been suggested that there is a critical window for estrogen replacement therapy (ERT) in postmenopausal women with Alzheimer’s disease (AD); however, supporting evidence is lacking. To address this issue, we investigated the effective period for estradiol (E2) treatment using a mouse model of AD. Four-month-old female APPswe/PSEN1dE9 (APP/PS1) mice were ovariectomized (OVX) and treated with E2 for 2 months starting at the age of 4 months (early period), 6 months (mid-period), or 8 months (late period). We then evaluated hippocampal neurogenesis, β-amyloid (Aβ) accumulation, telomerase activity, and hippocampal-dependent behavior. Compared to age-matched wild type mice, APP/PS1 mice with intact ovaries showed increased proliferation of hippocampal neural stem cells (NSCs) at 8 months of age and decreased proliferation of NSCs at 10 months of age; meanwhile, Aβ accumulation progressively increased with age, paralleling the reduced survival of immature neurons. OVX-induced depletion of E2 in APP/PS1 mice resulted in elevated Aβ levels accompanied by elevated p75 neurotrophin receptor (p75^NTR^) expression and increased NSC proliferation at 6 months of age, which subsequently declined; accelerated reduction of immature neurons starting from 6 months of age, and reduced telomerase activity and worsened memory performance at 10 months of age. Treatment with E2 in the early period post-OVX, rather than in the mid or late period, abrogated these effects, and p75^NTR^ inhibition reduced the overproliferation of NSCs in 6-month-old OVX-APP/PS1 mice. Thus, E2 deficiency in young APP/PS1 mice exacerbates cognitive deficits and depletes the hippocampal NSC pool in later life; this can be alleviated by E2 treatment in the early period following OVX, which prevents Aβ/p75^NTR^-induced NSC overproliferation and preserves telomerase activity.

## Introduction

The risk for Alzheimer’s disease (AD) is associated with age-related loss of ovarian hormones in women (Marongiu, 2019). Prospective and case-control studies have demonstrated that estrogen replacement therapy (ERT) can effectively reduce cognitive deficits in some but not all postmenopausal women (Espeland et al., 2004; Vedder et al., 2014). Growing evidence suggests that the effectiveness of ERT depends on its application during a critical period and a specific treatment duration (Espeland et al., 2004; Zandi et al., 2002). Experiments in aged nonhuman primates and rodents have revealed that 17β-estradiol (E2) treatment following ovariectomy can improve cognitive function (Baxter et al., 2018; Pollard et al., 2018) and alleviate cognitive decline in aged animals with long-term E2 deficiency (Luine, 2014). This critical treatment window may be related to changes in brain responsiveness to E2.

The generation and progression of β-amyloid (Aβ) accumulation in the brain is a pathologic hallmark of AD (Benilova et al., 2012). Many experiments have demonstrated the ability of Aβ to cause synaptic dysfunction and mature neuron death (He et al., 2019; Xu et al., 2012). Neurogenesis in the adult hippocampus involves the proliferation of NSCs and their terminal differentiation into neurons, which is important for preserving cognitive function (Ager et al., 2015). Improving hippocampal neurogenesis can ameliorate cognition in mouse models of AD (Xu et al., 2012), which exhibit a pathologic Aβ plaque burden accompanied by decreased hippocampal neurogenesis and impaired hippocampal-dependent spatial memory (Moon et al., 2014). A previous study has reported that Aβ impairs the proliferation of NSCs in the adult subventricular zone *via* p75^NTR^ (Sotthibundhu et al., 2009). Also, both *in vitro* and *in vivo* experiments have proven that the aggregated form of Aβ can inhibit telomerase activity (Wang et al., 2015), which has been proposed to preserve stem cell function by the promotion of resting stem cell proliferation and maintaining in telomere length (Boccardi and Paolisso, 2014). Postmenopausal women who received long-term estrogen therapy had longer telomeres than those who did not receive this therapy (Lee et al., 2005). There is increasing evidence that E2 can alter hippocampal structure and function (Frick et al., 2015), with long-term administration of E2 at physiologic doses reducing Aβ accumulation in 3xTg-AD mice (Carroll et al., 2007).

To explore the effective time window of E2 treatment for alleviating AD-associated cognitive impairment and the hippocampal mechanisms, we used female transgenic mice carrying mutant human *APPswe* and *PS1dE9* genes (APP/PS1 mice), in which the production of Aβ appears at approximately 4–5 months of age and dramatically accelerates during 6–8 months of age (Garcia-Alloza et al., 2006). In this study, 4-month-old female APP/PS1 mice were ovariectomized (OVX) to induce an acute decline in E2(surgical menopause), and then we evaluated the effects of E2 administered at different time points postsurgery on hippocampal neurogenesis, Aβ accumulation, and the causal link between the neuroprotective effect of E2 and its hippocampal-dependent memory performance.

## Materials and Methods

### Animals

APP/PS1 transgenic mice (Stock No. 34829) with a C57BL/6J × C3H hybrid background (Stock No. 004462) were obtained from Jackson Laboratory (Bar Harbor, ME, USA). APP/PS1 mice were maintained by crossing the hemizygotes with noncarrier siblings [later referred to as wild-type (WT) mice], and genotyped by polymerase chain reaction (PCR) analysis of genomic DNA from tail biopsies according to the instructions and our previous publication (Xu et al., 2012). Female hemizygous APP/PS1 mice aged 4–10 months and their WT littermates were used in this study. The present study was approved by the Institutional Animal Care and Ethical Committee of Nanjing Medical University (No. 2014-153), and conducted in accordance with the guidelines of the Institute for Laboratory Animal Research of the Nanjing Medical University. Mice were housed in plastic cages at 23 ± 2°C and 55% relative humidity with a 12:12 h light/dark cycle. Water and food were given *ad libitum*. Body weight was measured weekly.

### Ovariectomy and Drug Administration

#### Ovariectomy

**Figure 1:**
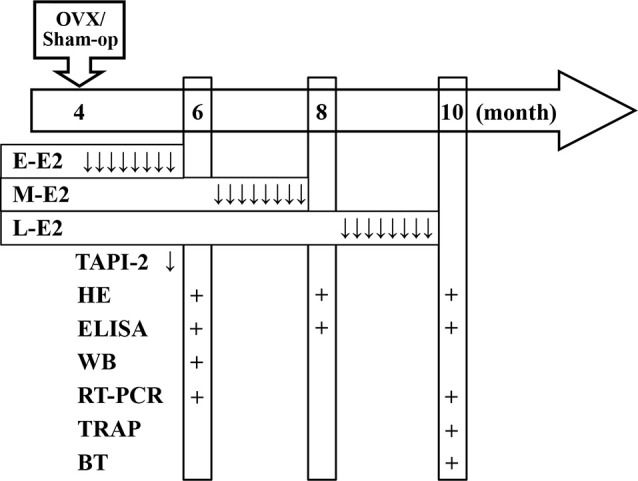
Time chart of the experimental procedure. Horizontal arrow indicates the mouse age (month). ↓, E2/TAPI-2 administration; +, time point of experiment examination; E-E2, early period; M-E2, mid period; L-E2, late period; HE, histological examination; WB, western blotting; BT, behavioral tests.

All surgeries were performed under ketamine (100 mg/kg)/xylazine (10 mg/kg; i.p.) anesthesia using aseptic conditions following institutional guidelines. Female mice in diestrus were bilaterally ovariectomized (OVX) at the age of 4 months (Figure 1). The dorsal surface was shaved and the vascular supply was ligated, then ovaries dissected out through bilateral incisions. Sham-operated (sham-op) control mice were subjected to surgery and ovaries were manipulated but left intact.

#### E2 Treatment

A pellet containing 0.18 mg of E2 (Innovative Research of America, Sarasota, FL, USA) was subcutaneously implanted at the following three time points: immediately after OVX, 2 months after OVX (6-month-old), or 4 months after OVX (8-month-old). The pellet continuously releases E2 for 60 days, resulting in serum levels of estrogen in the physiological range (Carroll et al., 2007). At the end of each period of E2 treatment, specimens were collected or behavioral tests were carried out (see time chart of the experimental procedure in Figure 1).

#### TAPI-2 Treatment

The p75^NTR^ metalloprotease inhibitor TAPI-2 (Sigma–Aldrich, St. Louis, MO, USA) was intracerebroventricular injected by the osmotic pump infusion 4 days before 5-bromo-2′-deoxyuridine (BrdU) injection (Figure 1). The mouse was placed in the stereotaxic instrument (Stoelting, Wood Dale, IL, USA), and a 7-day Alzet osmotic minipump (mean flow rate, 0.25 μl/h) containing TAPI-2 (50 mmol/l; Katakowski et al., 2007) or sterile saline was placed subcutaneously in the back. A brain infusion cannula connected to the pump that was positioned at right lateral ventricle (0.3 mm posterior, 1.0 mm lateral, and 2.3 mm ventral to Bregma).

### Histological Examination and Quantitative Evaluation

The mice were injected (i.p.) with BrdU (50 mg/kg; Sigma–Aldrich) twice daily at 6 h intervals for three consecutive days as previously described (Taniuchi et al., 2007). At 24 h after the last BrdU injection, the mice were anesthetized and perfused transcardially with cold phosphate-buffered saline (PBS) followed by 4% paraformaldehyde (PFA). Brains were post-fixed overnight in 4% PFA at 4°C and coronal section (40 μm) were cut using a vibrating microtome (Microslicer DTK 1500, Dousaka EM Company, Kyoto, Japan).

#### BrdU, Ki67 and Doublecortin (DCX) Immunostaining

Every 5th free-floating section (200 μm apart) was treated with 2 M HCl for 30 min and then with 3% hydrogen peroxide for 10 min at room temperature. The sections were incubated with PBS containing 0.3% Triton X-100 and 3% normal goat serum for 45 min, followed by overnight incubation at 4°C with mouse monoclonal anti-BrdU antibody (1:1,000; Millipore, Billerica, MA, USA) or mouse monoclonal anti-DCX antibody (1:500; Santa Cruz, CA, USA). After several PBS rinses, the sections were incubated in biotin-labeled goat anti-mouse IgG antibody (1:500; Santa Cruz) under agitation. Immunoreactivities were visualized based on avidin-biotin horseradish peroxidase complex (ABC Elite; Vector Laboratories, Inc., Burlingame, CA, USA) using 3,3′-diaminobenzidine (DAB; Vector Laboratories). For Ki67 immunostaining, the sections were incubated with rabbit monoclonal anti-Ki67 antibody (1:500; Abcam, Cambridge, UK) followed by Cy3-conjugated goat anti-rabbit IgG antibody (1:200, Jackson ImmunoResearch Laboratories, PA, USA).

#### Quantitative Evaluation of Immuno-Positive Cells

Immuno-positive BrdU (BrdU^+^) and DCX (DCX^+^) cells were assessed using a conventional light microscope (Olympus DP70, Japan) with a 40× objective. Images of Ki67-immunolabeled sections were observed using a fluorescence microscope (Olympus DP70, ×100). The total number of BrdU^+^ cells or Ki67^+^ cells was calculated from 12 sections and multiplied by 5 to obtain a total number for each mouse. The number of DCX^+^ cells in the subgranular zone (SGZ) was counted and divided by the length of SGZ to obtain the density of DCX^+^ cells (per micrometer; Sha et al., 2016).

#### Aβ Immunochemistry and Plaque Counting

The blocked sections were incubated in rabbit polyclonal anti-Aβ (1:300; Invitrogen, Carlsbad, CA, USA). Immuno-reactivities were visualized by the ABC method. Aβ-labeled plaques in the hippocampus with a diameter of more than twice the size of a neuronal cell body (the mean diameter is approximately 15 μm; Gilman et al., 2017; Weisenburger and Vaziri, 2018) were counted, and the number was multiplied by 5 to obtain the total number of plaques in the hippocampus (Carroll et al., 2007).

### Enzyme-Linked Immunosorbent Assay (ELISA)

Sandwich Aβ ELISAs were performed as previously described (Heikkinen et al., 2004). Briefly, hippocampal samples were dissected from the mice under deep isoflurane anesthesia and homogenized in 8× cold guanidine buffer (50 mM Tris-HCl, pH 8.0, 5 M guanidine-HCl). The samples and Aβ-peptides used as standards were diluted with reaction buffer containing protease inhibitors and AEBSF (Roche, Mannheim, Germany) to prevent degradation of Aβ and centrifuged at 16,000× *g* for 20 min at 4°C. The quantity of total protein in the supernatants was quantified by bicinchoninic acid (BCA) protein assay kit (Pierce Biotechnology Inc., Rockford, IL, USA). The total levels of hippocampal Aβ were quantified using human Aβ_42_ and Aβ_40_ELISA kits (Invitrogen #KHB3441 and #KHB3481, respectively) following the manufacturer’s instructions. Absorbance at 450 nm was measured with an automated microplate reader (ELx800 BioTek Inc., Winooski, VT, USA). Aβ_42_ and Aβ_40_ concentrations in the samples were standardized to total protein levels and are expressed in nanograms per milligram of total protein.

### Western Blotting Analysis

The brains were rapidly removed and the coronal brain slices (1 mm) were cut in ice-cold PBS. The dentate gyrus (DG) of the hippocampus was micro-dissected from the sections on dry ice and homogenized. The protein (50 μg) was separated by SDS-PAGE and transferred to membranes. The antibodies used were rabbit polyclonal anti-p75^NTR^ (1:200; Santa Cruz) and mouse monoclonal anti-β-actin (1:2,000; Millipore). Horseradish peroxidase-conjugated secondary antibodies were applied and the immunoreactive signals visualized with enhanced chemiluminescence detection system (Millipore). Protein band intensity was quantified using the image analysis software package (ImageJ; NIH Image, Bethesda, MD, USA) and is expressed as the ratio relative to β-actin level.

### Reverse Transcription-Polymerase Chain Reaction (RT-PCR)

Total RNA from DG regions was extracted using TRIzol reagent kit (Invitrogen). cDNA synthesis was performed using the Prime Script RT reagent kit (TaKaRa; Japan), according to manufacturer’s instructions. Briefly, 500 ng of RNA was used as a template to prepare cDNA. A 1 μl 20-fold diluted cDNA (each sample in triplicate) was then used for each run. The primer sets used for *p75*^NTR^, *GAPDH*, *TERT*, and β-*actin* were designed according to the previous publications (AlMatrouk et al., 2018; Zhang et al., 2019). PCR conditions for *TERT* and β-*actin* were 30 cycles of denaturation at 94°C for 45 s, annealing at 65°C for 45 s, and extension at 72°C for 45 s. PCR product of *TERT* was resolved by 1.5% agarose gel electrophoresis and visualized by ethidium bromide staining. The band intensities were determined using the image analysis software package (ImageJ), and β-*actin* gene expression was detected as the internal control. The other PCR products were assessed using an ABI Prism 7300 Sequence Detection System (Applied Biosystems, Foster City, CA, USA) in the presence of a fluorescent dye (SYBR Green I; TaKaRa). The relative expression of genes was determined using the 2^−ΔΔCt^ method, with normalization to *GAPDH* expression. The values were averaged from four sets of independent experiments and are expressed as a percentage relative to WT mice.

### Telomeric Repeat Amplification Protocol (TRAP) Assay

The telomerase activity was measured from fresh hippocampal DG regions using the TRAP_EZE_^®^ XL Telomerase Detection Kit (#S7707; Millipore), following the manufacturer’s instruction and previously described (An et al., 2017) with some modifications. All samples were analyzed in triplicate. Briefly, samples were resuspended in CHAPS lysis buffer and incubated on ice for 30 min. Each reaction contained 10 μl 5× TRAP_EZE_RT Reaction mix, 0.4 μl (2 U) Taq polymerase, 37.6 μl nuclease-free H_2_O, and 2 μl sample extract (500 ng/μl). The TSR8 control template was used to generate a standard curve. After a 30 min incubation at 30°C, the samples were amplified over 38 cycles at 94°C for 30 s 59°C for 30 s and 72°C for 1 min. The amplified telomerase products were quantified using a fluorescence plate reader (MQX200 BioTek Inc.). Telomerase activity was calculated from the ratio of telomerase products to an internal standard for each lysate (ΔFL/ΔR).

### Behavioral Tests

#### Y-Maze Test

Working memory performance was assessed by recording spontaneous alternation behavior (SAB) in a single-session Y-maze as described before (Zhang et al., 2019). The custom-made maze had wooden arms separated by 120° angle. Each arm was 30 cm long, 10 cm wide, and restricted by 20 cm high walls. The mice were placed at the center of the Y-maze and were allowed to freely explore for 8 min. The series of arm entries was recorded visually. An arm entry was considered to be complete when the mouse placed all four paws into a given arm. The percentage alternation was calculated as the ratio of actual alternations (the number of triads containing successive entries into the three arms) to the maximum possible alternations (the total number of arms entered minus 2).

#### Morris Water Maze (MWM) Test

Two days after Y-maze test, spatial learning and memory ability were evaluated with the MWM test (Zhang et al., 2019). A circular water tank made of white polypropylene (diameter = 120 cm; height = 45 cm) was prepared. The pool was filled to a depth of 33 cm with water (24 ± 1°C) made opaque by adding a small amount of non-toxic white paint. Swimming paths were analyzed using a computer system with a video camera (AXIS-90 Target/2; Neuroscience Inc., Tokyo, Japan). During the visible-platform training (2 days), a cylindrical dark-colored platform (7 cm in diameter) was placed 0.5 cm above the water surface with the position of the midpoint of one of the four quadrants. The mouse was randomly released from four different quadrants, respectively, and allowed to swim for 90 s. During the hidden-platform training (5 days), the platform was submerged 1 cm below the water surface opposite to the position in the visible-platform training. Four trials were conducted per day with a 30-min intertrial interval. Average swimming speed (m/s) and latency (s) to reach the platform were scored on all trials. On day 8 of training, a probe trial was carried out by removing the platform. The mouse was released from the opposite quadrant in which the platform was located in the hidden-platform training and allowed to swim for 90 s, and the percentage of time spent in the target quadrant was used to evaluate memory retention.

### Data Analysis/Statistics

All of the outcome analyses were carried out by the independent investigators blinded to the treatment conditions and mouse types. Data were retrieved and processed with the software Micro cal Origin 9.1 (Origin Lab Corp., Northampton, MA, USA). Group data were expressed as mean ± standard error (SE), and statistical analyses were performed using SPSS software, version 18.0 (SPSS Inc., Chicago, IL, USA). Experimental results were compared among treatment groups by analysis of variance (ANOVA) or Student’s *t*-test, followed by Bonferroni’s *post hoc* analysis for significant effects. *P* < 0.05 was considered statistically significant.

## Results

### Age-Related Changes in Hippocampal Neurogenesis in APP/PS1 Mice and WT Mice

**Figure 2:**
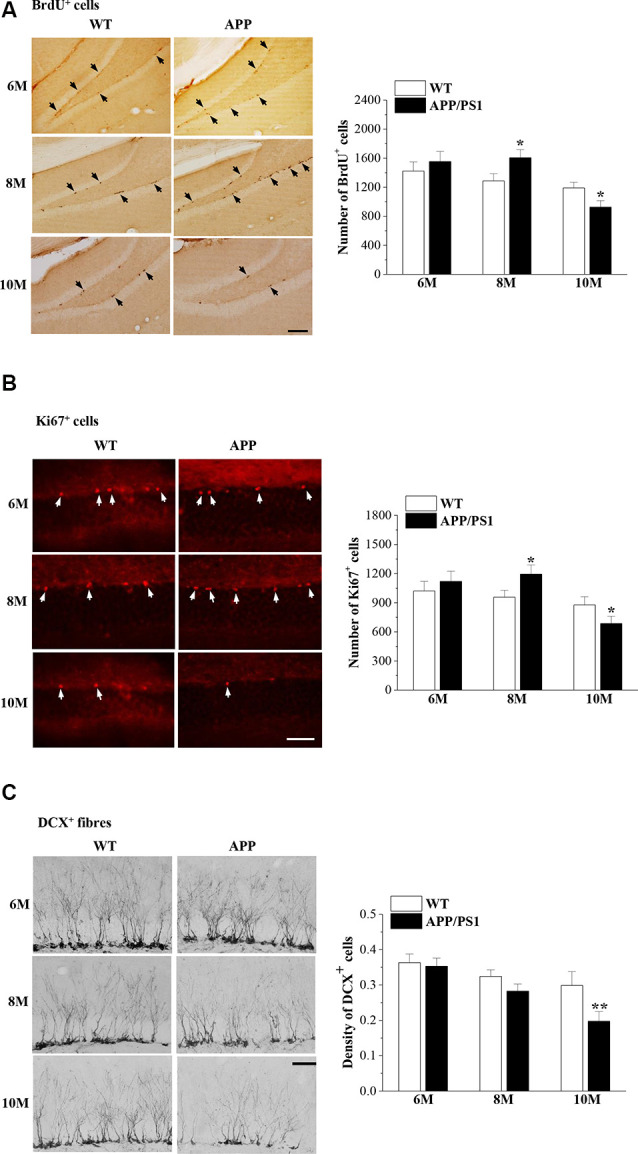
Abnormal neurogenesis in the dentate gyrus (DG) of APP/PS1 mice. **(A)** Representative images of 5-bromo-2′-deoxyuridine (BrdU) immunostaining in 6-month-old, 8-month-old, and 10-month-old APP/PS1 mice and age-matched wild-type (WT) mice (upper panels). BrdU^+^ cells are indicated by black arrows. Scale bar = 50 μm. The bar graphs show the mean number of BrdU^+^ cells in APP/PS1 mice and WT mice. *P < 0.05 vs. age-matched WT mice (Student’s *t*-test). **(B)** Ki67 immunostaining in 6-month-old, 8-month-old, and 10-month-old APP/PS1 mice and age-matched WT mice (upper panels). Ki67^+^ cells are indicated by white arrows. Scale bar = 50 μm. The bar graphs show the mean number of Ki67^+^ cells in APP/PS1 mice and WT mice. *P < 0.05 vs. age-matched WT mice (Student’s *t*-test). **(C)** Representative pictures of doublecortin (DCX) immunostaining in 6-month-old, 8-month-old, and 10-month-old APP/PS1 mice and age-matched WT mice (upper panels). Scale bar = 50 μm. The bars indicate the mean density of DCX^+^ cells in APP/PS1 mice and WT mice. **P < 0.01 vs. 10-month-old WT mice (Student’s *t*-test).

Our previous study reported that the proliferation of NSCs in the DG of male 8-month-old (8 m)-APP/PS1 mice is increased compared with that in age-matched male WT mice (Xu et al., 2012). In the present study, we used exogenous (BrdU) and endogenous (Ki67) markers of mitosis to examine the proliferation of NSCs and the microtubule-associated protein DCX to detect newly generated healthy neurons in female APP/PS1 mice and WT mice at the ages of 6 months, 8 months and 10 months (6m, 8m and 10m). Interestingly, we observed that the numbers of BrdU^+^ cells (*t*_(14)_ = −0.688, *P* = 0.503; Figure 2A) and Ki67^+^ cells (*t*_(14)_ = −0.685, *P* = 0.504; Figure 2B) in the SGZ of 6m-APP/PS1 mice were not significantly different from those of 6m-WT mice. Notably, the numbers of BrdU^+^ cells (*t*_(14)_ = −2.787, *P* = 0.015) and Ki67^+^ cells (*t*_(14)_ = −2.290, *P* = 0.038) in 8m-APP/PS1 mice were markedly higher than those in 8m-WT mice. In contrast, an obvious decline in the number of BrdU^+^ cells (*t*_(14)_ = 2.738, *P* = 0.016) or Ki67^+^ cells (*t*_(14)_ = 2.578, *P* = 0.022) was observed in 10m-APP/PS1 mice compared to 10m-WT mice. In addition, the density of DCX^+^ cells in 10m-APP/PS1 mice (*t*_(14)_ = 6.303, *P* < 0.001; Figure 2C), but not 6m-APP/PS1 mice (*t*_(14)_ = 0.309, *P* = 0.762) or 8m-APP/PS1 mice (*t*_(14)_ = 1.521, *P* = 0.151), was lower than that in age-matched WT mice. Thus, hippocampal NSC proliferation initially increases and then decreases while the survival of immature neurons decreases with age in APP/PS1 mice.

### Effects of E2 on Hippocampal Neurogenesis in APP/PS1 Mice and WT Mice

**Figure 3:**
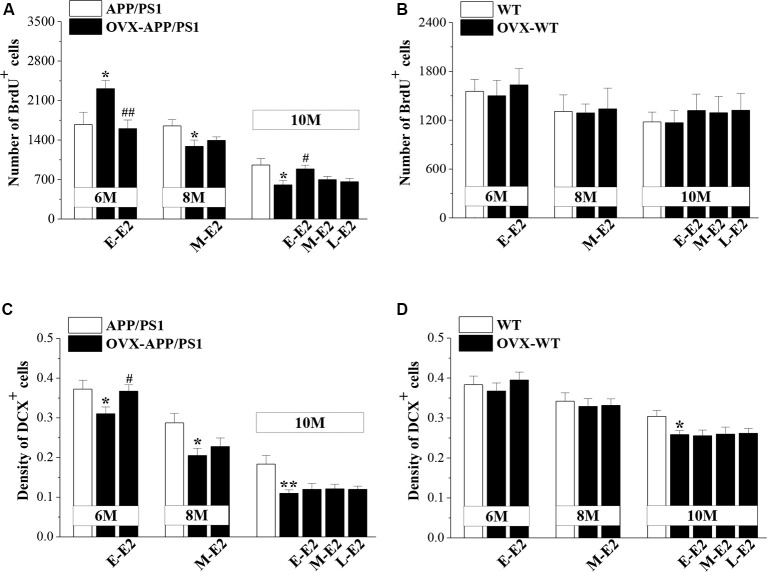
Influence of E2 on the proliferation of neural stem cells (NSCs) and the survival of immature neurons in the DG of APP/PS1 mice. **(A,B)** Bar graphs showing the numbers of BrdU^+^ cells in APP/PS1 mice or WT mice that were sham-operated (sham-op; open bars) or OVX (black bars). *P < 0.05 vs. age-matched sham-op APP/PS1 mice; ^#^*P* < 0.05 and ^##^*P* < 0.01 vs. age-matched OVX-APP/PS1 mice (two-way ANOVA). **(C,D)** The mean density of DCX^+^ cells in APP/PS1 mice or WT mice. *P < 0.05 and **P < 0.01 vs. age-matched sham-op mic; ^#^*P* < 0.05 vs. 6-month-old OVX-APP/PS1 mice (two-way ANOVA).

To test the effects of E2 on the age-related changes in NSC proliferation and neuron production in APP/PS1 mice, 4m-APP/PS1 mice and 4m-WT mice were bilaterally OVX and treated with E2 for 2 months at three different stages (early period: E-E2; mid period: M-E2; and late period: L-E2; Figure 1). Compared to sham-op 6m-APP/PS1 mice, OVX-6m-APP/PS1 mice showed an increase in the number of BrdU^+^ cells (*P* = 0.028, *n* = 8; Figure 3A), which was abrogated by the administration of E-E2 (*P* = 0.005, *n* = 8). However, OVX-8m-APP/PS1 mice (*P* = 0.040, *n* = 8) or OVX-10m-APP/PS1 mice (*P* = 0.023, *n* = 8) showed a significant decrease in the number of BrdU^+^ cells compared to the age-matched sham-op APP/PS1 mice. The number of BrdU^+^ cells was comparable between M-E2 treated OVX-8m-APP/PS1 mice and vehicle-treated OVX-8m-APP/PS1 mice (*P* = 0.292, *n* = 8). E-E2 treated OVX-10m-APP/PS1 mice exhibited more BrdU^+^ cells (*P* = 0.017, *n* = 8) than their vehicle-treated counterparts, but the M-E2 (*P* = 0.452, *n* = 8) and L-E2 (*P* = 0.588, *n* = 8) treatment groups had no change. The number of BrdU^+^ cells did not differ between OVX-WT mice and sham-op WT mice (*F*_(1,42)_ = 0.057, *P* = 0.813; Figure 3B), or between E2- and vehicle-treated OVX-WT mice (*F*_(3,56)_ = 0.399, *P* = 0.754) at these three ages.

OVX-6m-APP/PS1 mice (*P* = 0.044, *n* = 8; Figure 3C), OVX-8m-APP/PS1 mice (*P* = 0.018, *n* = 8) and OVX-10m-APP/PS1 mice (*P* = 0.007, *n* = 8) showed obvious loss of DCX^+^ cells when compared to the age-matched sham-op APP/PS1 mice. However, the density of DCX^+^ cells was unchanged in OVX-6m-WT mice (*P* = 0.654, *n* = 8; Figure 3D) and OVX-8m-WT mice (*P* = 0.701, *n* = 8), but was reduced in OVX-10m-WT mice (*P* = 0.023, *n* = 8) compared to age-matched sham-op WT littermates. The administration of E-E2 reversed the decrease in DCX^+^ cell density in OVX-6m-APP/PS1 mice (*P* = 0.032, *n* = 8), but E-E2 administration was ineffective in OVX-6m-WT mice (*P* = 0.401, *n* = 8). Furthermore, the administration of M-E2 did not alter the DCX^+^ cell density in OVX-8m-APP/PS1 mice (*P* = 0.161, *n* = 8) or OVX-8m-WT mice (*P* = 0.364, *n* = 8); and the decreases in DCX^+^ cell density in OVX-10m-APP/PS1 mice (*F*_(3,28)_ = 0.213, *P* = 0.887) and OVX-10m-WT mice (*F*_(3,28)_ = 0.478, *P* = 0.700) were insensitive to the administration of E2. These results indicate that E2 treatment in the early period post-OVX prevents excessive proliferation of NSCs and preserves immature neurons at the initiation of pathogenesis in APP/PS1 mice, which may have a certain protective effect on the proliferative capacity of NSCs at the advanced stages of the disease.

### Effects of E2 on Aβ Secretion and Deposition in APP/PS1 Mice

**Figure 4:**
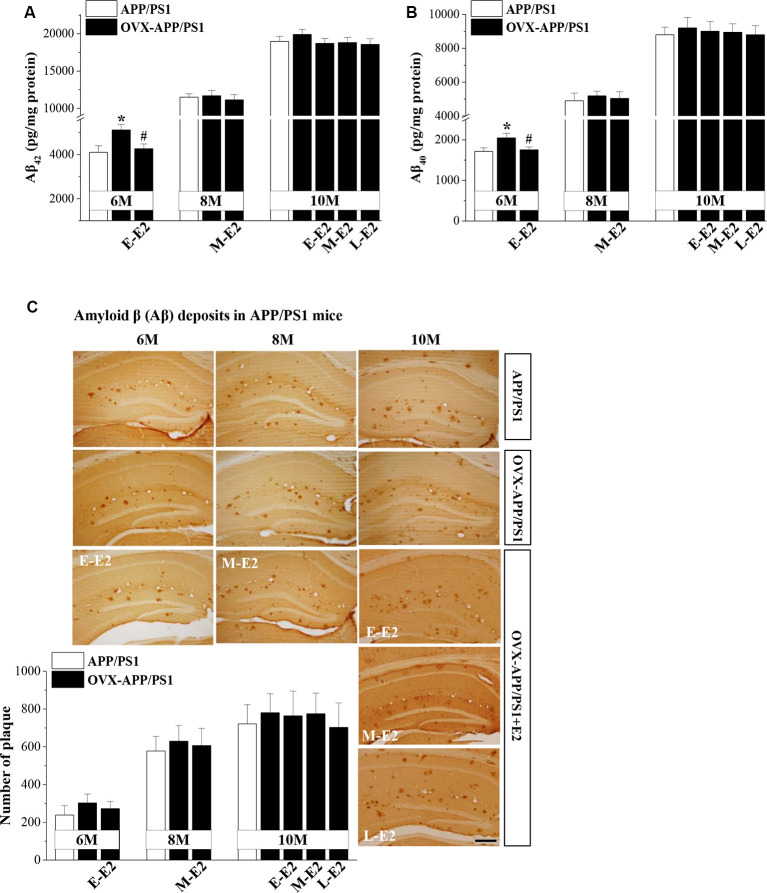
Age-related increase in Aβ accumulation and the influence of E2 on Aβ formation in the hippocampus of APP/PS1 mice. **(A,B)** Bar graphs showing the level of total Aβ_1–42_ or Aβ_1–40_in APP/PS1 mice that were sham-op (open bars) or OVX (black bars). *P < 0.05 vs. 6-month-old sham-op APP/PS1 mice; ^#^*P* < 0.05 vs. 6-month-old OVX-APP/PS1 mice (two-way ANOVA). **(C)** Representative images showing Aβ immunostaining in 6-month-old, 8-month-old, and 10-month-old APP/PS1 mice that were sham-op or OVX with vehicle or E2 treatment in the early period (E-E2), mid period (M-E2), or late period (L-E2). The data show the mean counts of Aβ deposits in the hippocampus of APP/PS1 mice. Scale bar = 200 μm.

To explore the mechanisms underlying E2-induced protection of neurogenesis in APP/PS1 mice, we evaluated the secretion and deposition of Aβ. OVX-6m-APP/PS1 mice showed elevated levels of Aβ_42_ (*P* = 0.025, *n* = 8; Figure 4A) and Aβ_40_ (*P* = 0.034, *n* = 8; Figure 4B) compared to sham-op 6m-APP/PS1 mice, which were reduced by the administration of E-E2 (Aβ_42_: *P* = 0.027, *n* = 8; Aβ_40_: *P* = 0.042, *n* = 8). However, OVX did not affect Aβ_42_ or Aβ_40_level in the hippocampus of 8m-APP/PS1 mice (Aβ_42_: *P* = 0.818, *n* = 8; Aβ_40_: *P* = 0.597, *n* = 8) or 10m-APP/PS1 mice (Aβ_42_: *P* = 0.365, *n* = 8; Aβ_40_: *P* = 0.604, *n* = 8). Furthermore, the administration of E2 in OVX-8m-APP/PS1 mice (Aβ_42_: *P* = 0.572, *n* = 8; Aβ_40_: *P* = 0.757, *n* = 8) or OVX-10m-APP/PS1 mice (Aβ_42_: *F*_(3,28)_ = 0.734, *P* = 0.540; Aβ_40_: *F*_(3,28)_ = 0.088, *P* = 0.966) had no effect on the level of Aβ_42_ or Aβ_40_. The number of Aβ plaques—another index of Aβ pathology—did not differ between OVX-APP/PS1 mice and the age-matched sham-op mice (*F*_(1,42)_ = 0.797, *P* = 0.377; Figure 4C) or between E2-treated OVX-APP/PS1 mice and the vehicle-treated groups at these three ages (*F*_(3,56)_ = 1.518, *P* = 0.220). The results indicate that E2 is beneficial for the hippocampi at the initiation stage of Aβ formation rather than at the more advanced stage of Aβ deposition.

### Involvement of p75^NTR^ in the Effects of E2-Induced Protection of Neurogenesis in APP/PS1 Mice

**Figure 5:**
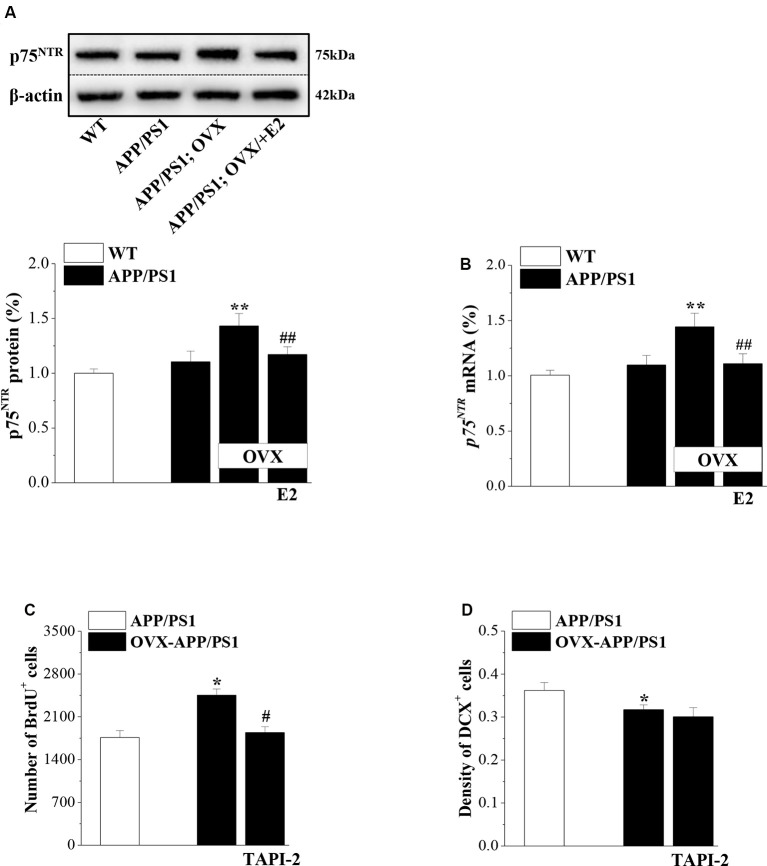
p75^NTR^ participates in E2-induced protection of NSC proliferation in APP/PS1 mice. **(A,B)** Influence of E2 on the level of p75^NTR^ in 6-month-old APP/PS1 mice. The upper panels represent representative blots of p75^NTR^. Bar graphs show the level of p75^NTR^ protein or *p75^NTR^* mRNA in APP/PS1 mice or OVX-APP/PS1 mice treated with vehicle or E2 for 2 months. **P < 0.01 vs. sham-op APP/PS1 mice; ^##^*P* < 0.01 vs. OVX-APP/PS1 mice (two-way ANOVA). **(C,D)** Influence of the p75^NTR^ inhibitor TAPI-2 on the excessive proliferation of NSCs and survival of immature neurons in the DG of 6-month-old APP/PS1 mice. The bar graphs show the number of BrdU^+^ cells and the density of DCX^+^ cells in APP/PS1 mice or OVX-APP/PS1 mice treated with vehicle or TAPI-2 for 7 days. *P < 0.05 vs. sham-op APP/PS1 mice; ^#^*P* < 0.05 vs. OVX-APP/PS1 mice (two-way ANOVA).

Activated p75^NTR^ ligands were shown to be involved in the neurogenic effect of Aβ (Sotthibundhu et al., 2009). To test the role of p75^NTR^ in the preservation of neurogenesis by E2 in APP/PS1 mice, we examined p75^NTR^ expression in 6-month-old mice that were OVX or sham-op at 4 months old and treated with E2 or vehicle for 2 months (Figure 1). Compared to 6m-WT mice, levels of p75^NTR^ protein (*P* = 0.082, *n* = 8; Figure 5A) and mRNA (*P* = 0.056, *n* = 8; Figure 5B) in 6m-APP/PS1 mice had a slight but insignificant increase. OVX-6m-APP/PS1 mice showed markedly higher levels of p75^NTR^ protein (*P* < 0.001, *n* = 8) and mRNA (*P* < 0.001, *n* = 8) than sham-op 6m-APP/PS1 mice. The application of E2 abolished the increases in p75^NTR^ expression in OVX-6m-APP/PS1 mice (*P* < 0.001, *n* = 8 for both protein and mRNA). We further used TAPI-2 to prevent the activation of p75^NTR^ and examined Aβ-impaired neurogenesis. Compared to vehicle-treated OVX-6m-APP/PS1 mice, the number of BrdU^+^ cells was significantly reduced in TAPI-2-treated OVX-6m-APP/PS1 mice (*P* = 0.014, *n* = 8; Figure 5C); however, the density of DCX^+^ cells was unchanged (*P* = 0.636, *n* = 8; Figure 5D). Thus, E2 deficiency results in elevated expression of p75^NTR^ and consequently, increased proliferation of hippocampal NSCs in APP/PS1 mice, but has no effect on the reduction of immature neurons.

### Effects of E2 on Telomerase Regulation in Aged APP/PS1 Mice

**Figure 6:**
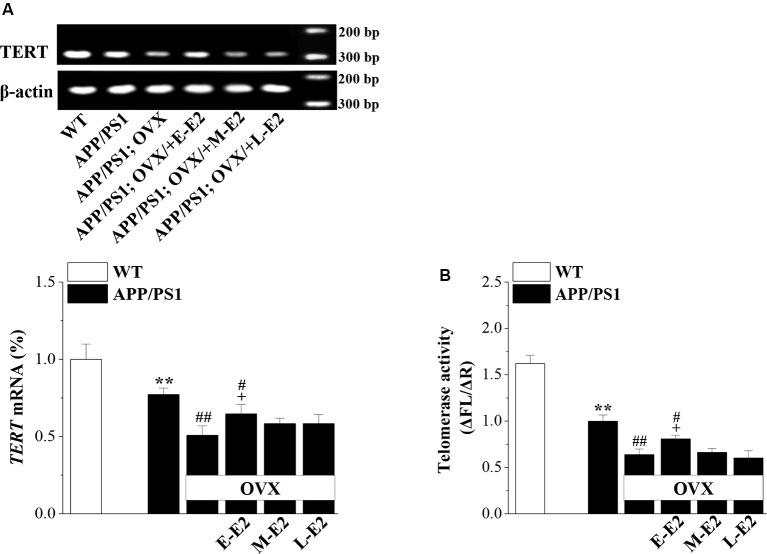
The administration of E2 in the early period post-OVX rescues the impaired telomerase activity in the DG in aged APP/PS1 mice. **(A)** Representative RT-polymerase chain reaction (PCR) analysis showing mRNA levels of *TERT* in 10-month-old APP/PS1 mice that were sham-op or OVX with vehicle or E2 treatment in the early period (E-E2), mid period (M-E2), or late period (L-E2). The bar graphs show the mRNA percentage level of *TERT*. **P < 0.01 vs. WT mice; ^#^*P* < 0.05 and ^##^*P* < 0.01 vs. sham-op APP/PS1 mice; ^+^*P* < 0.05 vs. OVX-APP/PS1 mice (two-way ANOVA). **(B)** Bar graphs showing the quantification of telomerase activity by telomeric repeat amplification protocol (TRAP; ΔFL/ΔR). **P < 0.01 vs. WT mice; ^#^*P* < 0.05 and ^##^*P* < 0.01 vs. sham-op APP/PS1 mice; ^+^*P* < 0.05 vs. OVX-APP/PS1 mice (two-way ANOVA).

Telomerase, a reverse transcriptase, is required for embryonic and adult neurogenesis (Lobanova et al., 2017). To investigate whether telomerase is implicated in the E2 mediated protection of NSCs in APP/PS1 mice, we analyzed the levels of TERT and telomerase activity in 10-month-old APP/PS1 mice. Compared to 10m-WT mice, 10m-APP/PS1 mice displayed significantly lower *TERT* mRNA level (*P* < 0.001, *n* = 8; Figure 6A) and telomerase activity (*P* < 0.001, *n* = 8; Figure 6B). However, *TERT* level (*P* < 0.001, *n* = 8) and telomerase activity (*P* < 0.001, *n* = 8) were markedly lower in OVX-10m-APP/PS1 mice than in sham-op 10m-APP/PS1 mice, but were increased by the administration of E-E2 (mRNA: *P* = 0.011, *n* = 8; activity: *P* = 0.037, *n* = 8; the administration of E2 is shown in Figure 1). However, compared to sham-op 10m-APP/PS1 mice, the *TERT* level (*P* = 0.016, *n* = 8) and telomerase activity (*P* = 0.032, *n* = 8) in OVX-10m-APP/PS1 mice that received E-E2 treatment were not completely restored. The administration of M-E2 or L-E2 had no effect on *TERT* expression (all *P* > 0.05, *n* = 8) or telomerase activity (all *P* > 0.05, *n* = 8) in OVX-10m-APP/PS1 mice. These results indicate that telomerase activity declines in the Aβ-impaired hippocampus, which is exacerbated by E2 deficiency; however, this is reversed by early period E2 treatment.

### Effects of E2-Induced Protection of Neurogenesis on Cognitive Deficits in Aged APP/PS1 Mice

**Figure 7:**
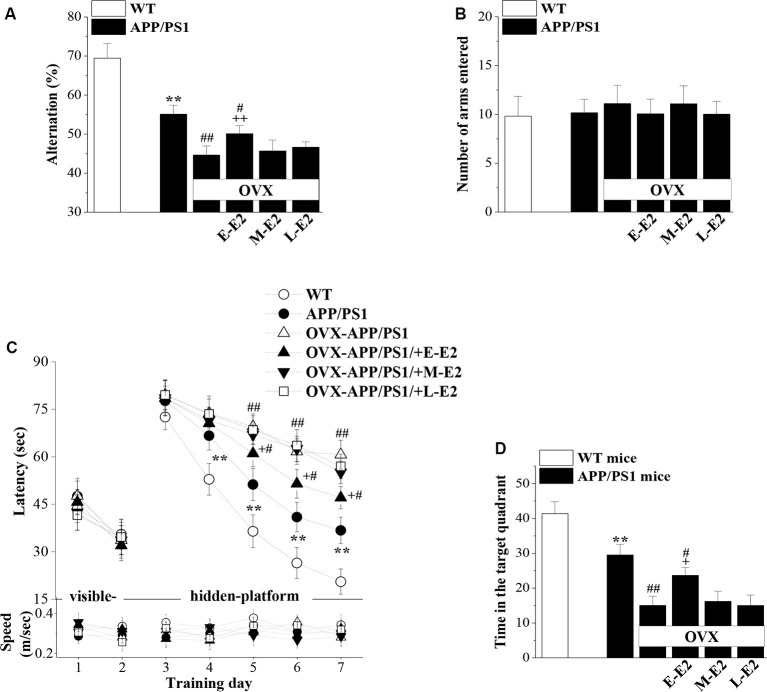
The administration of E2 in the early period post-OVX alleviates the cognitive deficits in aged APP/PS1 mice. **(A,B)** Influence of E2 treatment in the early period (E-E2), mid period (M-E2), or late period (L-E2) on working memory as assessed by the Y-maze test in 10-month-old APP/PS1 mice. The bar graphs show the spontaneous alternation ratio (%) and the total number of arm entries in the Y-maze test of APP/PS1 mice. **P < 0.01 vs. WT mice; ^#^*P* < 0.05 and ^##^*P* < 0.01 vs. sham-op APP/PS1 mice; ^++^*P* < 0.01 vs. OVX-APP/PS1 mice (two-way ANOVA). **(C)** Spatial learning and memory were assessed by the Morris water maze (MWM). Each point represents the mean latency (sec) to reach the platforms (upper panel) and the swimming speeds (m/s; bottom panel). **P < 0.01 vs. WT mice; ^#^*P* < 0.05 and ^##^*P* < 0.01 vs. sham-op APP/PS1 mice; ^+^*P* < 0.05 vs. OVX-APP/PS1 mice (repeated measure ANOVA). **(D)** Probe trial test. The bars represent the percentages of swimming time spent in the platform quadrant. **P < 0.01 vs. WT mice; ^#^*P* < 0.05 and ^##^*P* < 0.01 vs. sham-op APP/PS1 mice; ^+^*P* < 0.05 vs. OVX-APP/PS1 mice (two-way ANOVA).

Adult hippocampal neurogenesis has been implicated in the cognitive deficits that are a signature abnormality of AD (Gonçalves et al., 2016). Thus, it is critically important to assess whether E2-mediated protection of neurogenesis in APP/PS1 mice is linked to improvements in cognitive function. Hippocampal-dependent learning and memories were evaluated by the Y-maze and MWM at the age of 10 months (Figure 1). The SAB rate in the Y-maze, which is regarded as a measure of short-term working memory, was remarkably reduced in 10m-APP/PS1 mice compared with that in 10m-WT mice (*P* < 0.001, *n* = 10; Figure 7A). Moreover, OVX-10m-APP/PS1 mice showed a distinctly decreased rate compared to sham-op 10m-APP/PS1 mice (*P* < 0.001, *n* = 10). Central to our analysis was the observation that E-E2 administration partly restored the alternation rate (*P* = 0.003, *n* = 10) but did not completely reverse the impairment in working memory in OVX-10m-APP/PS1 mice relative to sham-op 10m-APP/PS1 mice (*P* = 0.026, *n* = 10). Additionally, the administration of M-E2 and L-E2 had no effect on the SAB rate in OVX-10m-APP/PS1 mice (all *P* > 0.05, *n* = 10). The rate in 10m-WT mice was unaffected by E2 deprivation and E2 treatment (data not shown). To exclude the possibility that the changes in SAB performance were caused by differences in motility, we quantified the number of arms entered by mice in the Y-maze. As shown in Figure 7B, there was no significant effect of genotype (*F*_(1,54)_ = 0.582, *P* = 0.449), ovariectomy (*F*_(1,54)_ = 0.005, *P* = 0.944) or E2 treatment (*F*_(3,54)_ = 1.191, *P* = 0.322) on the number of arm entries.

In the MWM, the visible-platform on days 1–2 was used to examine search behavior or visual acuity, and the hidden-platform on days 3–7 was used to evaluate hippocampal-dependent spatial cognitive function. During acquisition, mice showed no difference in the latency to reach the visible-platform based on genotype (*F*_(1,108)_ = 0.356, *P* = 0.552; Figure 7C), OVX (*F*_(1,108)_ = 0.000, *P* = 0.996), or E2 treatment (*F*_(3,108)_ = 0.371, *P* = 0.774). On days 4–7 of training, the latency to reach the hidden platform in 10m-APP/PS1 mice was significantly increased compared to that in the 10m-WT mice (all *P* < 0.05, *n* = 10), but there was no difference in swim speed between these two groups (all *P* > 0.05, *n* = 10). Furthermore, compared to sham-op 10m-APP/PS1 mice, OVX-10m-APP/PS1 mice required a longer time to find the hidden platform on days 5–7 (all *P* < 0.05, *n* = 10) with no change in swimming speed (all *P* > 0.05, *n* = 10). Interestingly, the increased latency on days 5–7 of training in OVX-10m-APP/PS1 mice was reversed by the administration of E-E2 (day 5: *P* = 0.015, *n* = 10; day 6: *P* = 0.016, *n* = 10; day 7: *P* = 0.012, *n* = 10), but not by the administration of M-E2 or L-E2 (all *P* > 0.05, *n* = 10). However, the administration of E-E2 alone in OVX-10m-APP/PS1 mice did not completely recover the prolonged latency compared to sham-op 10m-APP/PS1 mice (day 5: *P* = 0.048; day 6: *P* = 0.011; day 7: *P* = 0.013). No significant difference was found in the swimming speed between E2- and vehicle-treated OVX-10m-APP/PS1 mice (all *P* > 0.05).

A probe trial was carried out 24 h after the last day of training, and the percentage of time spent in the target quadrant was measured to evaluate the strength of the memory trace. As shown in Figure 7D, 10m-APP/PS1 mice spent less time in the target quadrant than 10m-WT mice (*P* < 0.001). OVX-10m-APP/PS1 mice showed much shorter swimming time in the target quadrant than sham-op 10m-APP/PS1 mice (*P* < 0.001). In addition, the swimming time in the target quadrant was longer for E-E2–treated OVX-10m-APP/PS1 mice than for vehicle-treated OVX-10m-APP/PS1 mice (*P* = 0.021), but shorter than for sham-op 10m-APP/PS1 mice (*P* = 0.023). The performance in the target quadrant by OVX-10m-APP/PS1 mice was unaltered by the administration of M-E2 or L-E2 (*P* > 0.05 in each group). Taken together, these results imply that E2 alleviates cognitive deficits in aged APP/PS1 mice, but only when administered during the early period post-OVX.

## Discussion

Consistent with the evidence supporting the existence of a critical period for the benefit of ERT on cognitive function in postmenopausal women with AD (Vedder et al., 2014), here, we showed in AD model mice that E2 treatment in the early, but not in the late period post-OVX, prevented cognitive decline. This involved inhibition the excessive proliferation of hippocampal NSCs by suppression of Aβ-induced overexpression of p75^NTR^ in young APP/PS1 mice, and preservation of telomerase activity and proliferation capability of NSCs in aged APP/PS1 mice. The positive effects on cognition were evidenced by better scores in the Y-maze and MWM tests.

APP/PS1 mouse models develop AD pathology at young age, enabling the study of exogenous E2 efficiency on cognitive performance under varying conditions of ovarian function. Whilst OVX can be used to study the consequences of menopause in women, the procedure induces a rapid gonadal hormone decline in contrast to the progressive decline associated with natural menopause in women. Nevertheless, a recent review has suggested that OVX results in a “blank ovarian hormone state,” which is an ideal model to evaluate the effects of ovarian hormone deprivation and the outcomes of subsequent exogenous hormone treatments on the brain (Koebele and Bimonte-Nelson, 2016). Indeed, epidemiological data regarding women who underwent OVX before the onset of natural menopause indicate a long-term increased risk of AD (Rocca et al., 2010) and furthermore ERT efficacy is similar in both natural menopause and oophorectomy in women, depending on the timing of treatment initiation (Mosconi et al., 2004; Maki et al., 2019). Given that female mice continue to experience hormonal cycles with age (Hung et al., 2003), the use of OVX can be considered an effective model to mimic human/primate menopause in mice.

The results presented here are in line with previous studies demonstrating that the proliferation of hippocampal NSCs is increased at the Aβ plaque initiation stage while subsequently decreased at the Aβ plaque progressive stage in the same mouse model as ours (Taniuchi et al., 2007; Xu et al., 2012), indicating a similar “bell-shaped” proliferation curve. However, the survival of immature neurons declined faster with age in APP/PS1 mice, showing a steeper curve than that in WT mice. The turning point seems to be at the age of occurrence of massive Aβ deposits. Meanwhile, in agreement with earlier findings, our results showed progressive exacerbation of Aβ pathology in APP/PS1 mice from the age of 6–10 months (Garcia-Alloza et al., 2006). Our previous report demonstrated that Aβ impairs the survival of immature neurons by downregulating the PI3K-Akt-mTOR signaling pathway (Li et al., 2010). The sensitivity of the adult brain to Aβ was found to be reduced after 10 months of age in mice, indicating an age-dependent decrease in the neurogenic effect of Aβ (Sotthibundhu et al., 2009). Therefore, the bell-shaped curve may be attributed to the gradual desensitization of the nervous system to Aβ and depletion of the NSC pool; the decline in NSC proliferation could contribute to the progressive reduction in new neuron production in aged APP/PS1 mice. However, another AD mouse model, APP/PS1 mice on a C57BL/6J background exhibit seizure activity at the age of 3–4 months, and show different hippocampal neurogenesis from our model (Unger et al., 2016). These heterogeneities of phenotypes may be attributable to genetic background and inter-animal variability. Additionally, although APP/PS1 transgenic mouse models have been commonly used in AD research, the pathogenesis in these models has distinctions from sporadic AD patients such as lacking of widespread neurodegeneration and none developed neurofibrillary tangles (NFTs; Drummond and Wisniewski, 2017). Further study is needed to determine the critical period of E2 on an ideal model contains both plaques and NFTs.

Our results showed that estrogen deficiency caused an increase in NSC proliferation at the Aβ initiation stage and a decrease at the Aβ progressive stage, showing a leftward shift of the proliferation curve. The survival of immature neurons showed a sharper decline in APP/PS1 mice than in WT mice. E2 treatment in the early period post-OVX prevented the over-proliferation of NSCs and increased the immature neuron. The relationship between E2 and hippocampal neurogenesis has been controversial. Exogenous E2 administration enhances hippocampal cell proliferation by activating estrogen receptor, whereas long-term E2 deficiency has no effect on cell proliferation in the DG in female rats or mice (Tanapat et al., 2005; Galea et al., 2006; Lagace et al., 2007). Importantly, E2 treatment at the early period post-OVX decreased the levels of Aβ. According to previous studies, E2 treatment for 3 months reduces the enhanced Aβ accumulation in OVX-3xTg-AD mice (Carroll et al., 2007). E2 treatment can increase Aβ clearance *via* regulation of β-secretase (BACE1) activity and expression in APP23 mice (Li et al., 2013). A 2-month administration of docosahexaenoic acid-enriched phospholipids inhibits the generation and accumulation of Aβ by suppressing BACE1 expression in senescence-accelerated prone eight mice (Wang et al., 2019). These evidences may explain the effect of E2 on Aβ accumulation. Investigations into the relationship between Aβ and hippocampal neurogenesis have proven that exogenous and endogenous Aβ promote the proliferation of NSCs in the subventricular zone *via* p75^NTR^ (Sotthibundhu et al., 2009; Zheng J. Y. et al., 2017), and Aβ inhibits the endocytosis of p75^NTR^ in cholinergic basal forebrain neurons (Ovsepian et al., 2014). p75^NTR^ is shown to stimulate the proliferation of cultured neurospheres by interacting with several cell-cycle regulatory proteins (Provenzano et al., 2011). Our findings showed that the levels of Aβ and p75^NTR^ were elevated by OVX, both prevented by E2 treatment in the early period post-OVX; blocking p75^NTR^ activation reduced NSC proliferation without affecting the survival of immature neurons. Thus, the increased survival of immature neurons by early-period E2 treatment might be related to the reduction in Aβ levels. One study has demonstrated that the deletion of the p75^NTR^ gene suppresses Aβ production in APP/PS1 mice (Wang et al., 2011). Lower p75^NTR^ mRNA and protein levels have been observed following chronic E2 treatment (Hasan et al., 2005). Thus, there are multiple levels of interaction between E2, Aβ, and p75^NTR^. Combined with these observations, our results indicate that early period E2 treatment blocked the hyperproliferation of NSCs by inhibiting Aβ-induced p75^NTR^ expression, and alleviated the depletion of the NSC pool in aged APP/PS1 mice, thereby abolishing the leftward overproliferation curve. In accordance with the E2-induced preservation of NSC proliferation in aged OVX-APP/PS1 mice, we found that telomerase activity was reduced in aged APP/PS1 mice, which was exacerbated by OVX and rescued by early-period E2 treatment post-OVX. These observations imply that changes in telomerase activity are accompanied by altered NSC number in the DG. Telomerase is expressed at high levels in NSCs and neural progenitor cells in the adult brain (Liu et al., 2018), and Aβ aggregates are shown to inhibit telomerase activity in senescent human cells (Wang et al., 2015). This implies that the decrease in telomerase activity in aged APP/PS1 mice may be related to the Aβ accumulation. However, we observed that there were no significant differences in Aβ levels between aged APP/PS1 mice, OVX-APP/PS1 mice and E2 treated OVX-APP/PS1 mice, raising the possibility that the NSC pool provides a supply of telomerase. Additionally, pharmacologic induction of TERT is found to increase the expression of nerve growth factor and brain-derived neurotrophic factor (BDNF) while protecting neurons from oxidative stress and the cytotoxic effects of Aβ (Baruch-Eliyahu et al., 2019), implying an interaction between the NSC pool and telomerase.

Interestingly, E2 had beneficial effects on neurogenesis and Aβ levels in APP/PS1 mice only when administered during the early period post-OVX. Our findings in this regard can be explained by previous studies showing that the neuroprotective actions of E2 are dependent on time since OVX. The administration of E2 within 3 months, but not delayed 10 months post-OVX, has efficacy in rats (Walf et al., 2009). It has been proposed that the responsiveness of the brain to hormones declines after an extended period of hormone depletion (Hamilton et al., 2011). E2 deficiency or administration after the progressive stage did not affect Aβ levels or the plaque numbers in APP/PS1 mice. Although some studies suggest that estrogen deprivation for 3 months could enhance the deposition of Aβ in APPswe transgenic mice that underwent OVX at 4 weeks old (Levin-Allerhand and Smith, 2002), estrogen treatment after a similar interval following OVX has no effect on Aβ levels in mice that underwent OVX at an older age (Heikkinen et al., 2004). This is in general agreement with earlier work demonstrating that long-term E2 deficiency increased Aβ production at the initiation stage of amyloid pathology but has no effect on Aβ aggregation and plaque formation (Heikkinen et al., 2004; Levin-Allerhand et al., 2002). E2 had no effect on hippocampal neurogenesis in WT mice, although 10-month-old OVX-WT mice showed fewer immature neurons, which is supported by reports that E2 does not affect neurogenesis in normal mice (Lagace et al., 2007).

In addition to E2-mediated effects on hippocampal NSC proliferation, we also observed that E2 treatment in the early period post-OVX partially alleviated cognitive deficits in aged APP/PS1 mice. Adult neurogenesis depends on the function and stable numbers of NSCs. Continuous neurogenesis in the DG is essential for maintaining normal hippocampal-dependent cognitive behavior. The hippocampal mechanism of context encoding is considered to depend on a subset of immature adult-born neurons in the DG (Adlaf et al., 2017). NSC transplantation restores spatial learning ability in 18-month-old 3xTg-AD mice by elevating hippocampal BDNF level (Blurton-Jones et al., 2009), and stimulating NSC proliferation in 7-month-old 3xTg-AD mice by activating the PI3K-AKT-GSK3β-Wnt pathway results in increased cognitive function (Zheng R. et al., 2017). The relationship between NSC and synaptic resistance to Aβ toxicity has been confirmed by *in vivo* and *in vitro* experiments: a larger pool of NSC is associated with reduced Aβ binding to the synapse, while transgenic ablation of endogenous NSC increases synaptic Aβ binding (Micci et al., 2019). Factors secreted during the proliferation of hippocampal NSCs create a favorable microenvironment for the proper functioning of the nervous system (Lee et al., 2007). Moreover, E2 treatment in young OVX mice can lead to a long-lasting enhancement of classical estrogen response element-dependent gene activation in the hippocampus, the effects of which can persist even after cessation of E2 treatment (Pollard et al., 2018).

A recent multimodality brain imaging study has shown that declines in circulating estrogens are a major risk factor for female-specific brain AD abnormalities, and suggests that the therapeutic window of ERT for AD preventive interventions in women might be early in menopause (Rahman et al., 2020). The present study provides evidence that the early period following OVX is the critical window for E2 treatment to alleviate cognitive deficits in aged APP/PS1 mice. E2 likely acts by preventing depletion of the hippocampal NSC pool and preserving basal neurogenesis. Our findings reveal the mechanisms underlying the cognitive efficacy of E2 treatment, especially as related to a critical window after hormone declines, and provide experimental evidence that can guide clinical decision-making for the initiation of ERT in postmenopausal women, particularly those with or at risk of developing AD.

## Data Availability Statement

The raw data supporting the conclusions of this article will be made available by the authors, without undue reservation.

## Ethics Statement

The animal study was reviewed and approved by Institutional Animal Care and Ethical Committee of Nanjing Medical University.

## Author Contributions

SS conceived and designed the experiments. YQ and DA performed the experiments. WX and XQ analyzed the data. XW contributed reagents and materials. LiC, LeC, and SS drafted the manuscript. All authors contributed to the article and approved the submitted version.

## Conflict of Interest

The authors declare that the research was conducted in the absence of any commercial or financial relationships that could be construed as a potential conflict of interest.
